# A Novel Technique Using Fluorescent Ureteral Catheter and Flexible Ureteroscope for Safe Laparoscopic Fenestration of Lymphocele after Kidney Transplantation

**DOI:** 10.1155/2022/9948425

**Published:** 2022-02-07

**Authors:** Takanori Sekito, Motoo Araki, Koichiro Wada, Kasumi Yoshinaga, Yuki Maruyama, Takuya Sadahira, Satoshi Katayama, Takehiro Iwata, Shingo Nishimura, Tomoko Sako, Kohei Edamura, Yasuyuki Kobayashi, Toyohiko Watanabe

**Affiliations:** ^1^Department of Urology, Okayama University Graduate School of Medicine, Dentistry and Pharmaceutical Sciences, Okayama 7008558, Japan; ^2^Department of Urology, Shimane University Faculty of Medicine, Izumo, Shimane 693-0021, Japan

## Abstract

Laparoscopic fenestration of a postrenal transplant lymphocele is associated with a risk of renal hilar vessel and ureteral injury. Consequently, determination of the incision line is difficult. We describe a case of a 73-year-old man with postrenal transplant lymphocele who underwent a laparoscopic fenestration. We report a surgical video containing a new technique of laparoscopic fenestration using a fluorescent ureteral catheter in combination with a flexible ureteroscope. The combination of a fluorescent ureteral catheter and flexible ureteroscope during surgery enabled us to determine the incision line safely and accurately. Intraoperative real-time visualization of the lymphocele and ureter using a fluorescent ureteral catheter and a flexible ureteroscope is safer than conventional methods for laparoscopic fenestration. To the best of our knowledge, this is the first report of this novel technique.

## 1. Introduction

Postrenal transplant lymphocele is not a rare complication of renal transplantation and may contribute to postrenal acute renal failure [1, 2]. Laparoscopic fenestration is one of the treatment options for lymphocele. However, it has a risk of renal hilar vessel or ureteral injury; therefore, determining the incision line during surgery is difficult.

The near-infrared ray catheter (NIRC™) fluorescent ureteral catheter (Nippon Covidien, Ltd., Tokyo, Japan) is a novel catheter containing fluorescent material that can be visualized under near-infrared irradiation. It provides the necessary anatomical information for avoiding ureteral damage and ensuring safer surgery [3–5].

Herein, we verified the usefulness of combining a fluorescent ureteral catheter with a flexible ureteroscope. To the best of our knowledge, this is the first case report of a novel, safe technique for laparoscopic fenestration of a lymphocele after kidney transplantation by using a fluorescent ureteral catheter and flexible ureteroscopy.

## 2. Case Presentation

A 73-year-old man with end-stage renal disease due to renal sclerosis and diabetic nephropathy underwent a living donor kidney transplantation from his wife after five years of hemodialysis. The patient was discharged on postoperative day 21 with a serum creatinine (Cr) level of 1.71 mg/dL.

**Figure 1:**
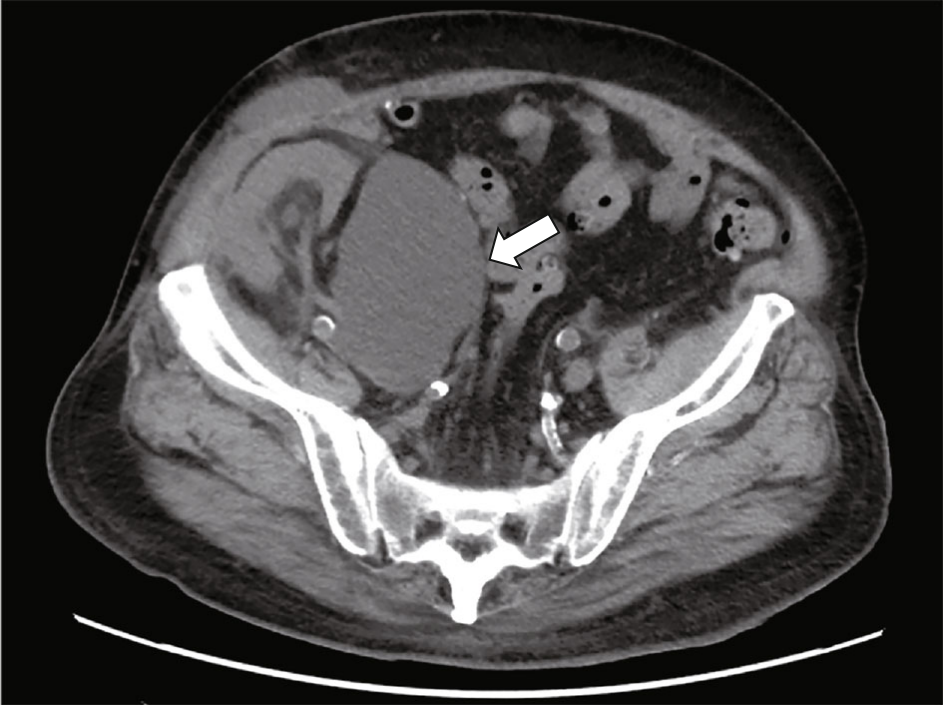
Computed tomography (CT). A lymphocele (16 cm × 8 cm × 6 cm) on the left side of the transplanted kidney in the right iliac fossa (white arrow) is detected.

On postoperative day 29, the Cr level rose to 2.32 mg/dL with appearance of right lower extremity swelling. Computed tomography (CT) showed a lymphocele on the left side of the transplanted kidney in the right iliac fossa (Figure 1). The lymphocele caused mild hydronephrosis and compression of the right common iliac and femoral veins. A CT-guided lymphocele drainage catheter was placed in the lymphatic cyst on postoperative day 34, but there was little improvement. Although lymphangiography was performed twice on postoperative days 41 and 82 for lymphatic vessel occlusion, there was no improvement. Then, the patient underwent a laparoscopic fenestration.

### 2.1. Operative Intervention

A 6 Fr NIRC™ fluorescent ureteral catheter was placed in the transplanted kidney by rigid cystoscopy in the lithotomy position.

The VISERA ELITE2 system (Olympus, Tokyo, Japan) was used to perform laparoscopic surgery with real-time infrared observation. Three ports were used for laparoscopic fenestration. After the laparoscope was inserted, the drainage catheter was removed under a guidewire. A flexible ureteroscope (URF-V2 (Olympus, Tokyo, Japan)) was inserted over the guidewire to observe the lymphocele cavity from the inside.

**Figure 2:**
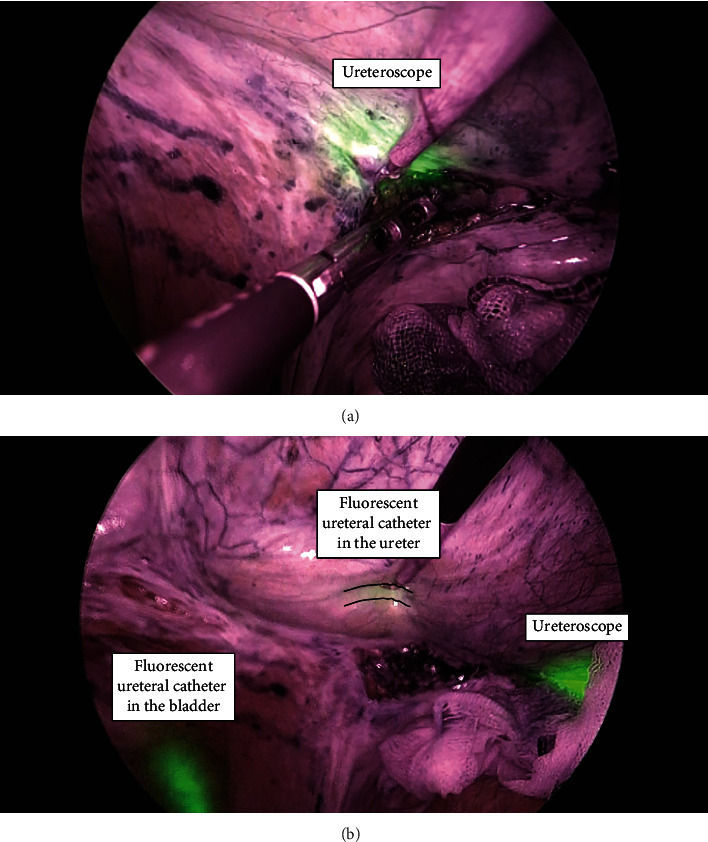
Intraoperative imaging in near-infrared mode. (a) The light source of the ureteroscope appears green. The thickened lymphocele wall is incised with a cautery hook while appropriately checking the light source of the ureteroscope in real time. (b) The fluorescent ureteral catheter in the bladder can be easily observed due to the green fluorescence. By compressing the cyst from the peritoneal cavity using forceps, the fluorescent catheter in the ureter can be safely checked (broken line). The light source of the ureteroscope is also visible as it appears green.

The lymphocele was identified from the peritoneal cavity, and the peritoneal wall around the incision site of the lymphocele was observed. The ureter of the transplanted kidney was in contact with the lymphocele; therefore, the incision line was chosen carefully. Observation using the ureteroscope was relatively easy due to the lumenization of the lymphocele. We verified the light source of the ureteroscope and marked the line of incision on the peritoneum by laparoscopic observation. In the near-infrared mode, we confirmed that the ureteral catheter in the bladder and the light source of the ureteroscope glowed green. The thickened lymphocele wall was incised with cold scissors and a cautery hook while appropriately checking the light source of the ureteroscope in real time (Figure 2(a)) till the incision reached the lymphocele cavity. In the near-infrared mode, we could safely identify the fluorescent catheter in the ureter by compressing the cyst from the peritoneal cavity using forceps (Figure 2(b)). A guidewire was inserted through the ureteroscope and held with laparoscopic forceps in the peritoneal cavity to suspend the lymphocele wall, which ensured safe incision of the lymphocele wall with a cautery hook. A total of 4 cm of the lymphocele wall was incised. A drain was placed near the incision from within the lymphocele and peritoneal cavity at the end of the surgery. The operative time was 210 minutes, and the laparoscopic part lasted for 138 minutes, with minimal estimated bleeding loss.

### 2.2. Postoperative Course

The postoperative course was uneventful, and the patient was discharged on postoperative day 6. The follow-up CT scan at 4 months showed no recurrence of the lymphocele with a baseline Cr level of 1.78 mg/dL.

## 3. Discussion

During laparoscopic fenestration of a postrenal transplant lymphocele, the combination of a fluorescent ureteral catheter and flexible ureteroscope enabled us to determine the incision line safely and accurately.

Lymphocele can occur in 0.6% to 51% of patients as a complication after kidney transplantation [6]. Clinical symptoms of symptomatic lymphocele include abdominal pain, lower urinary tract symptoms due to external compression by the lymphocele, worsening renal function caused by transplant ureteral obstruction, and ipsilateral lower extremity swelling due to venous obstruction. The cause of the leakage is considered to be either the lymphatic ducts in the recipient's iliac vessels or in the kidney graft [7, 8]. Saidi et al. suggested that a careful ligation of the severed lymphatic ducts of the kidney graft, which can be procured laparoscopically, may decrease the lymphatic complications after kidney transplantation [8]. Another report demonstrated that minimization of external iliac artery dissection and firm ligation of the lymph trunk may be effective in preventing persistent lymphatic leakage [9].

Laparoscopic fenestration is an efficient treatment for symptomatic lymphoceles with a recurrence rate between 4 to 8%, which is lower than that of other treatments such as aspiration, percutaneous drain placement, and sclerotherapy [2, 10]. Since the laparoscopic approach has a substantial risk of injury to vital structures such as the renal pelvis, ureter, bladder, or iliac vessels [7], determining the incision line can be challenging. However, open surgery has a higher rate of complications compared to laparoscopic surgery [10]. Real-time percutaneous ultrasonographic guidance is considered essential for successful laparoscopic surgery [1]. Recently, the fluorescent navigation technique has provided another option for ensuring safer laparoscopic surgery.

There have been some reports on the use of fluorescent ureteral catheters to avoid ureteral injury during laparoscopic colorectal surgery or total laparoscopic hysterectomy [11, 12], but not during laparoscopic fenestration in kidney transplant recipients. The use of these fluorescent ureteral catheters has the advantage of visualization of the ureter in real time, thus, preventing ureteral injury [13].

Indocyanine green (ICG) emits near-infrared fluorescence [3, 14]. The fluorescent ureteral catheter uses a fluorescent material with fluorescence wavelengths similar to those of ICG, and it can be visualized using a near-infrared camera. Besides, the light source of the ureteroscope is also visible and appears green. Since the normal light of the ureteroscope partly contains a fluorescent wavelength region, this region shows a fluorescence similar to the fluorescent ureteral catheter.

In conclusion, to the best of our knowledge, we performed the world's first laparoscopic fenestration using a fluorescent ureteral catheter in combination with a flexible ureteroscope for a postrenal transplant lymphocele. Intraoperative real-time visualization of the lymphocele and ureter using a novel fluorescent ureteral catheter and flexible ureteroscope is safer than conventional methods for laparoscopic fenestration.

## Data Availability

The data used to support the findings of this study are included within the article.

## Abbreviations

Cr: Serum creatinine
CT: Computed tomography
ICG: Indocyanine green
NIRC™: Near-infrared ray catheter.

## Supplementary Materials

Supplementary Materials The online version contains supplementary material available. Additional file 1: video of the laparoscopic fenestration. https://drive.google.com/file/d/1jEJcdMjL7bM9v7JdIiWsLqD2AbVwHCKh/view?usp=sharing
