# Supplementary material for: A Novel Technique Using Fluorescent Ureteral Catheter and Flexible Ureteroscope for Safe Laparoscopic Fenestration of Lymphocele after Kidney Transplantation
Source: Case Rep Transplant. 2022 Feb 7;2022:9948425. doi: 10.1155/2022/9948425 (PMC8844347; doi:10.1155/2022/9948425)
Supplement: Supplementary Materials — The online version contains supplementary material available. Additional file 1: video of the laparoscopic fenestration. https://drive.google.com/file/d/1jEJcdMjL7bM9v7JdIiWsLqD2AbVwHCKh/view?usp=sharing [file 9948425.f1.docx]

**Supplementary Information**

The online version contains supplementary material available.

**Additional file 1.** Video of the laparoscopic fenestration.

[**https://drive.google.com/file/d/1jEJcdMjL7bM9v7JdIiWsLqD2AbVwHCKh/view?usp=sharing**](https://drive.google.com/file/d/1jEJcdMjL7bM9v7JdIiWsLqD2AbVwHCKh/view?usp=sharing)
